# Effects of driver compensatory behaviour on risks of critical pedestrian collisions under simulated visual field defects

**DOI:** 10.1371/journal.pone.0231130

**Published:** 2020-04-09

**Authors:** Jieun Lee, Makoto Itoh

**Affiliations:** 1 Department of Risk Engineering, Graduate School of System and Information Engineering, University of Tsukuba, Tsukuba, Ibaraki, Japan; 2 Faculty of Engineering, Information and Systems, University of Tsukuba, Tsukuba, Ibaraki, Japan; Tongii University, CHINA

## Abstract

Compensatory behaviour is regarded as a helpful strategy to mediate drivers’ deteriorated hazard perception ability due to visual field defects. However, helpfulness of compensatory behaviour for drivers with advanced visual field defects has largely unexplored. This study aims to clarify the effectiveness and limitation of compensatory head movements in critical situations where included pedestrians stepping off a sidewalk under the simulation of advanced visual defects. 18 healthy-sighted drivers participated the data collection that was conducted in a driving simulator under three driving conditions: (1) without visual impairment, (2) with visual impairment and not performing active compensation, and (3) with visual impairment but performing active compensation. The result showed that active compensation led quick accelerator and brake response times, reducing the risk and number of pedestrian collisions. The active compensation led a decrease in the number of non-responses to hazardous pedestrians compared to while driving not performing compensation. However, the compensation could not reduce the number of pedestrian collisions to those of healthy-sighted drivers. Compensatory viewing behaviour contributed to improved driving performance as well as has limits to lead driving performance like healthy-sighted drivers. Developing driver assistance systems and practical compensatory strategies concerning the degrees of impairment and traffic conditions may provide opportunities to improve driving safety deteriorated hazard perception for visually impaired drivers.

## Introduction

Vision is an essential component for driving [1]. Good visual functions–i.e. visual field, visual acuity or night vision, are necessary to appropriately recognise traffic situations [2]. Among these functions, visual field is particularly problematic for driving safety compared to others. Whereas the effect of low visual acuity is weak for motor vehicle collisions [3], an impaired visual field by optic diseases, such as glaucoma or retinitis pigmentosa affects driving safety, including a crash-involvement [4–7]. The symptoms of such diseases develop quite slowly. Consequently, patients tend to be not aware of its severity and how it affects their driving [8]. Therefore, accounting for problems what visually impaired drivers encompasses and how to support such drivers are critical for traffic safety.

There are numerous studies to look into the relationship between visual field defects and driving safety. Johnson and Keltner [4] investigated accident risk between drivers with binocular visual field defects and normal visual field, and showed that the former was twice as likely to be involved in a traffic accident as the latter. Racette and Casson [9], on the other hand, described that the extent of visual field defects does not affect driving performance. Inconsistently, the increased likelihood of patients being involved in accidents over that of drivers with no visual field defects was observed regardless of the extent of visual field defects [10]. The higher the likelihood of accidents, the higher the degrees of a patient’s defect. Patients with advanced visual field defects but with good central visual field are likely to be involved in motor vehicle collisions [11]. Given the relationship, it seems reasonable that visual field loss yields a higher risk of traffic accidents in comparison with drivers with normal vision.

Visual field defects lead a variety of difficulties associated with driving safety. There are few findings that visual field impairment may not degrade driving performance–i.e. lane-keeping performance [12] or driving straight ahead [13]. However, a myriad of studies has reported the difficulties–e.g. driving within lane positions [14], coping with the sudden appearance of a pedestrian or vehicle [2, 15–16], an oncoming vehicle turned right crossing the driver’s path at intersection [17], immediate reaction to divided attention [18], or obstacle avoidance [18–21]. The difficulty due to visual field defects is closely related to a driver’s hazard perception; thus it is reported as one of the causes of traffic accidents [12, 15, 22].

Hazard perception is a key aspect determining driving performance and traffic safety. Timely as well as appropriate hazard perception is expected in dynamically changing driving situation, however, drivers may not shift their attention from the forward direction to other peripheral stimuli when their visual field is defected. The visual defects do not allow drivers to detect both static and moving pedestrian and to cope with moving objects [23–25]. Even for drivers with mild-to-moderate visual field defects, deteriorated visual fields cause a difficulty in detecting peripheral obstacles or hazard [15]. Hence, the deterioration of peripheral visual field cannot be considered in isolation from drivers’ hazard perception ability [12, 26–27].

Compensation has been consistently reported as one of the strategies of mediating some of the problems in detecting traffic hazards (e.g. [12, 28–30]). Compensatory eye movement leads to faster reaction to stimulus and lower traffic risk compared to patients who do not applied compensation [31]. Earlier studies have also shown that increasing exploratory eye and head movements allows such drivers to detect traffic hazards successfully [32–37]. A study of compensation that assessed driving performance of patients with visual field defects found that effective compensation types depend on visual field defects patterns–e.g. head movements and reducing vehicle speed are efficient in cases of peripheral and central visual field defects respectively [38]. An increased number of exploratory behaviour–i.e. eye and head movements is associated with successful collision avoidance [24, 35, 39–40].

Despite driver compensation being positioned to help enhance driving performance, compensation can, in certain contexts, be seen as an efficient strategy for improving traffic safety. Unlike inconsistent findings in terms of differences in driving performance between drivers with mild and moderate visual defects [9–10], it is clear that moderate or advanced visual field defects lead increased risk of unsafe driving [17, 41]. That is, the extent of the visual field defects is a factor influencing traffic safety. Many of studies showing the effects of compensation are limited to analyses of eye movements under mild-to-moderate visual field defects (e.g. [36, 38]). However, the relationship between compensatory behaviour and driver’s hazard perception with respect to advanced visual defects has not been studied extensively. Previous studies reported that normal monocular visual field extends to approximately 90° superiorly, 70° inferiorly, and 60° nasally from a fixation [42–43]. However, patients with advanced visual field defects have only approximately 15° central visual field with the loss of peripheral visual field [44]. Given that saccades under advanced visual field defects could not guide driver’s attention to peripheral stimuli, drivers with advanced defects are more susceptible to miss peripheral stimuli when making eye movements than head movements [33]. In addition, these are insufficient to address effects on hazard experience or the amount of compensation and driving performance quantitatively [29, 32]. Thus, it is necessary to reveal how compensation is effective at detecting peripheral critical targets during driving as well as to what extent compensation can improve driving safety in critical situations.

The aim of the current study is to examine the effects of compensatory behaviour of drivers under visual field defects on avoiding unexpected traffic hazards during simulated driving. The main hazardous events were designed in accordance with the previous findings that patients with visual field defects have difficulty in avoiding the sudden appearance of a pedestrian or other vehicle from the sides [2, 16, 38], and that pedestrian collision is one of the most frequent types of traffic accidents [45]. We hypothesised that performing frequent head movements leads to a certain level of driving performance–i.e. quick response to a pedestrian’s presence, reducing the number of collisions with the pedestrian–but even if driver compensation is applied as much as possible, compensation is inadequate to allow visually impaired drivers to improve driving performance to the levels of healthy-sighted drivers.

## Method

### Participants

Eighteen participants (8 females, mean age = 25.3 years old, SD = 6.7 years, range = 18–32, mean driving days per week = 5.5 days) were recruited from advertisements and were reimbursed JPY 820 per hour for taking part in the study. Eligibility criteria were as follows: having a valid Japanese driver’s license, driving daily with an eyesight better than 20/30, and a clear medical history with no diagnosis of optic diseases–e.g. glaucoma or retinitis pigmentosa. This research complied with the University of Tsukuba’s ethics code and was approved by the ethical review board (Reference Number 2014R63). Informed consent was obtained from each participant.

### Apparatus

A fixed driving simulator was used for the experiment. The simulator system comprised a steering wheel, a fixed driving seat, and accelerator and brake pedals. The simulator projected a driving scene on the wall in front of the drivers, resulting in a horizontal and vertical field-of-view of 70° and 30° respectively. To prevent changing their field of view and affecting their driving performance, the participants were asked to not change their position. The driving simulator limited the maximum vehicle speed to 50 km/h.

**Fig 1:**
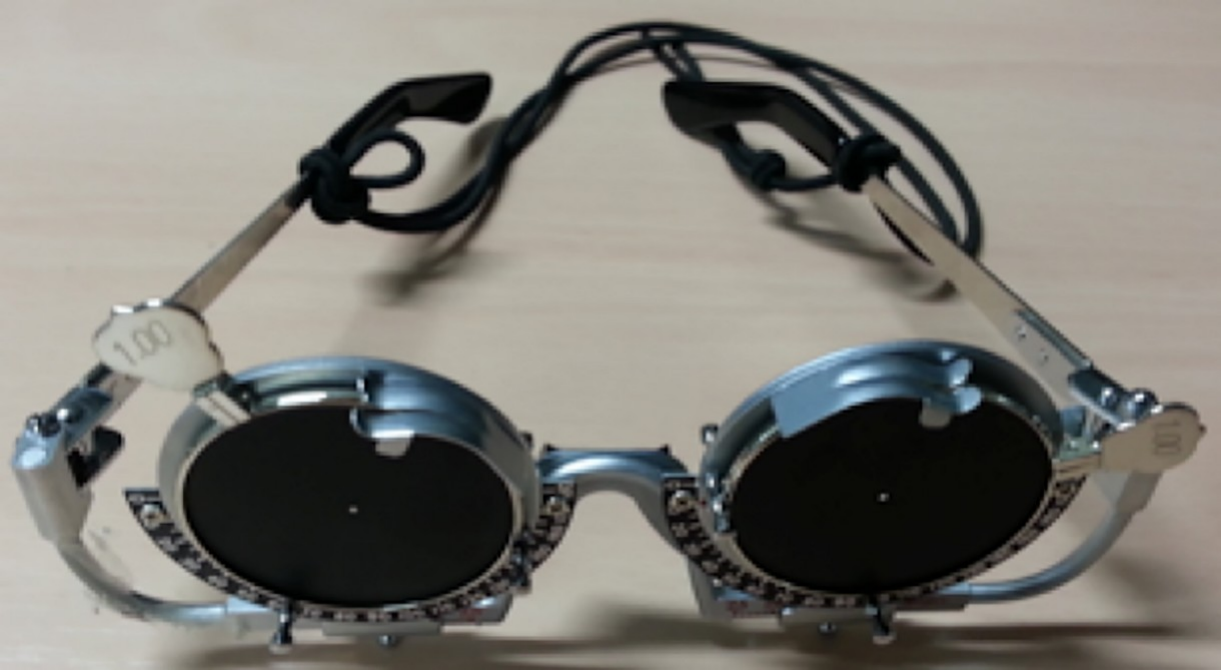
Eyeglass for visual field contraction.

**Fig 2:**
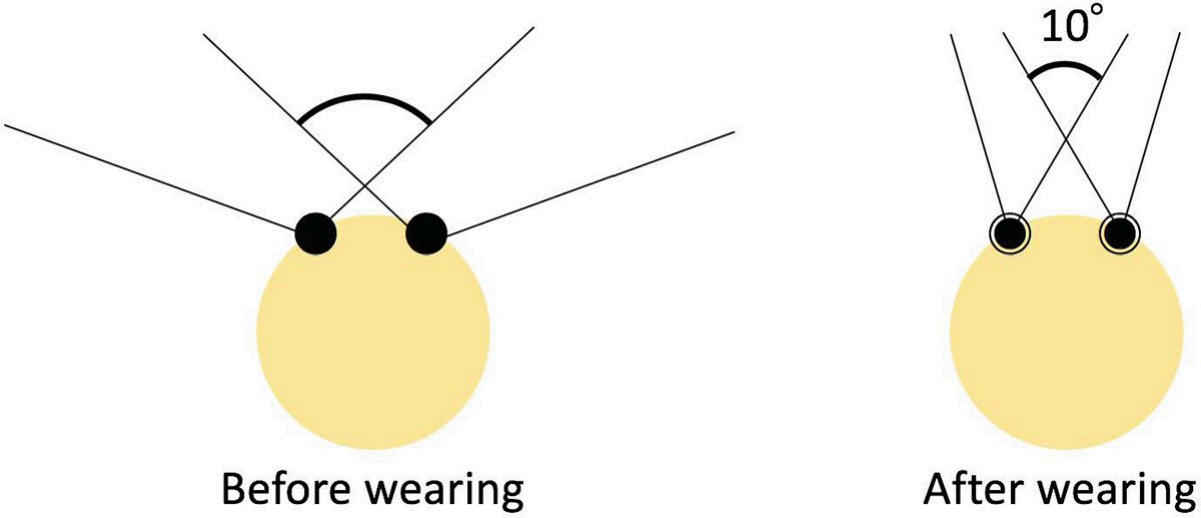
Participants’ view before and after wearing the eyeglass.

To imitate advanced peripheral visual field impairment, a special eyeglass was used during the experiment [20, 46–47]. It comprised a frame with a 1-mm pinhole lens (Fig 1) that contracts the participants’ binocular visual field angle to approximately 10° (Fig 2). A manual visual field tester not only measured the participants’ monocular visual field between 15 to 100° (nasally, superiorly, and inferiorly) but also assessed whether or not they put the eyeglass on appropriately. Participants were required to see a front mirror and not to move their eye to follow moving a white point that was moved by an experimenter. The experimenter made the participants aware of how narrow their visual field was when compared to their usual visual capability as well as confirmed whether the eyeglass was appropriately equipped for the constriction or not by using the tester. In addition, a video camera and a gyroscope were used to collect compensatory behaviour data. The gyroscope was mounted on a cap worn by all participants during each trial. The video camera was mounted in front of the driver. The gyroscope was updated at 50 Hz, whereas the logging of simulation data, such as speed, coordinates of the vehicle and the hazardous object, and input for the accelerator and brake pedals, was updated at a frequency of 10 Hz.

### Experimental conditions

A within-subject design was used to investigate the effect of compensation on reducing the risk of collisions with pedestrians. Each participant was tested under three driving conditions: driving without visual field defects (Baseline), driving with visual field defects and compensation (WC), and driving with visual field defects and without compensation (WoC). The order of the conditions was counterbalanced across participants. In the Baseline condition, participants drove without the eyeglass simulating visual field defects and could move their head freely. In the WC, they drove with the eyeglass generating simulated visual impairment and were required to follow instructions that asked them to actively make compensatory head movements. In the WoC, all participants drove with the eyeglass and were required to follow instructions that asked them not to perform compensation.

### Driving task and scenario

**Fig 3:**
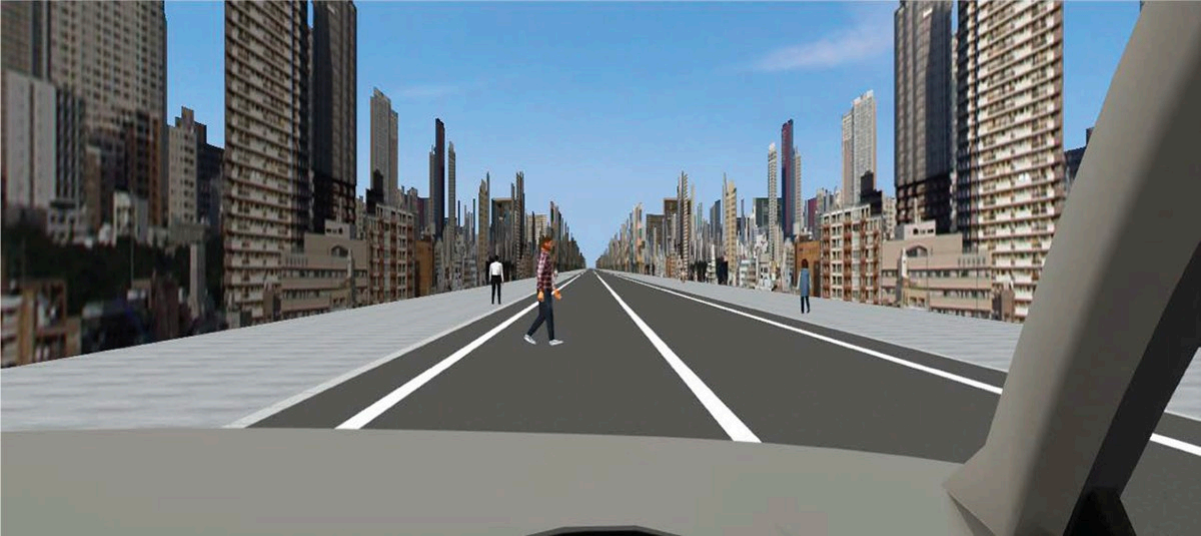
Hazardous driving scene.

During the experiment, the participants drove in the left-hand lane of a straight city while complying with the traffic laws and adhering to the speed limit of 50 km/h. As long as they pressed the accelerator pedal, the vehicle maintained a speed of 50 km/h; in order to maintain a constant speed, they were asked to continuously press the accelerator pedal during each session. During each trial, drivers experienced ten hazardous events in which a pedestrian unexpectedly rushed into the driving lane from the left sidewalk (Fig 3), and dummy events, including an oncoming right-turning car. The order of the main hazardous events was randomised, making it difficult for the participants to expect when and where the hazards would occur.

Prior to performing each trial, the participants were instructed on how hazardous events, such as pedestrian collisions and collisions with oncoming-turning vehicles, may cause a traffic accident. The participants could operate the accelerator and brake pedals when they felt that the situation was dangerous, and it corresponded to driver perception and recognition of hazards. In the current study, participants were not allowed for moving the steering wheel to react to the critical situation, but were rather required to release the accelerator and press the brake.

### Procedure

Upon arrival, a participant was presented with a written overview of the experiment and a consent form. After reading the overview and signing the consent form, the participant completed a test drive with no hazardous traffic events to familiarise him/her with the driving simulator. During the test drive, the participant was asked to drive until he/she became accustomed to the simulator. Prior to the start of each trial, the participant was instructed on the driving task and given a set of written instructions. After the explanation, a manual visual field tester was used to examine the monocular visual field of the participant. By using the tester, the participant became aware of how narrow his/her visual fields were. All participants practiced under each driving condition, and the practice continued until they felt accustomed to each driving condition. All practice drives contained hazardous events that were similar to the events during the actual trial. The practice sessions lasted approximately 2 min each. Next, the participants drove a trial, each of which lasted approximately 3 min. The time differed depending upon the number of times a driver coped with hazardous events–successful avoidance of the hazard resulted in longer trials. In total, the participants experienced three practice and three trial drives. Lastly, the participants filled out a questionnaire on their demographics, driving history, and opinion regarding each trial. They also took a break after each trial. The entire experiment lasted for approximately 2 h.

### Dependent variables

The number of compensatory head movements was counted to verify whether the participants followed the instructions. We judged drivers’ head movements from side to side while they scouted a traffic environment. We utilized two ways to count the number of head movements. It was both manually coded by the experimenter from a video (subjective count) and calculated from the gyroscope data which calculated by low pass filter (objective count).

The accelerator response time (s; ART) is defined as the time that elapses between the moment the pedestrian moves off the sidewalk and the driver’s complete release of the accelerator pedal. Brake response time (s; BRT) is defined as the time that elapses between the moment the pedestrian moves off the sidewalk and the driver’s initial application of the brake pedal. Two measures indicate drivers’ perception and recognition of the pedestrian respectively. The shorter the response time, the quicker the drivers were able to perceive and recognise the pedestrian. Time to collision (TTC) (s), which represents the risk of a collision with the pedestrian, served as a dependent variable. TTC was calculated using the distance between the host vehicle (*D*_*hy*_) and the pedestrian (*D*_*py*_) on the Y axis, as well as the speed of the host vehicle (*V*_*h*_) based on the accelerator response time (see Eq 1).

The number of pedestrian collisions was calculated during the experiment and re-examined through a comparison with the operation data from the driving simulator. In addition, subjective ratings on a 7-point scale were collected to investigate the difficulty of each trial for the participants. They were asked to respond the following question: “To what extent do you think the trial was difficult? (1 = not at all; 7 = absolutely)”.

$$Time\;to\;collision=\frac{D_{hy}-D_{py}}{V_{h}}$$ (1)

**Fig 4:**
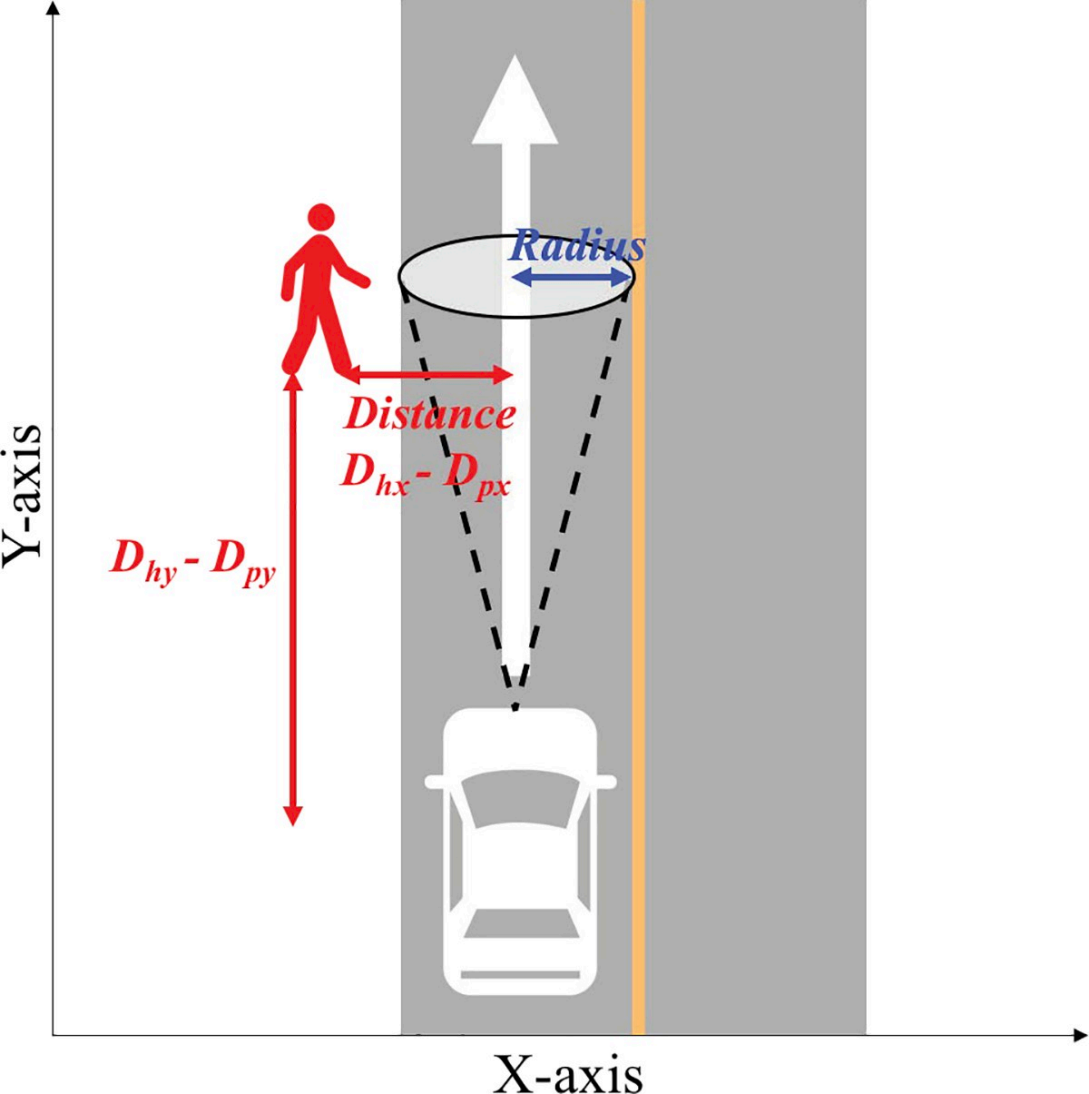
Relationship between radius and distance between the host vehicle and the pedestrian.

The radius (m), distance between the vehicle and pedestrian (m), and amount of compensatory head movements (°) served as dependent measures to examine the effects of compensation. According to CIE (International Commission on Illumination), at a viewing distance of 50 cm, a 10° field of view would be a diameter 8.8 cm circle. This study uses this concept to examine driver detection of hazardous objects. *Radius* represents the participant’s field of view under tubular visual field defects. While approaching a hazardous pedestrian on the Y axis (*D*_*hy*_*−D*_*py*_), the radius decreased and was calculated by Eq 2. Taking *Distance* between the host vehicle and pedestrian on the X axis (*D*_*hx*_*−D*_*px*_) and the radius, the range of compensation was used to estimate whether the driver was able to detect the pedestrian while applying compensation or not. Fig 4 shows the relationship between the distance and radius. The time point when distance (*D*_*hx*_*−D*_*px*_) exceeds the radius indicates that the hazard is out of driver’s sight.

$$Radius=0.088*(D_{hy}-D_{py})$$ (2)

### Statistical analyses

Correlation analyses were conducted to validate the subjectively and objectively counted number of compensatory head movements in the Baseline and WC condition. There was no analysis for the WoC condition because drivers did not move their head under the WoC. The correlation analysis examined the relationship between the number of compensatory head movements and collisions. In addition, the drivers were also divided into two groups based on the number of compensatory head movements: low- and high-compensation (half from bottom and top respectively), and independent-samples t-test was performed to analyse difference in the number of pedestrian collisions.

We employed One-way ANOVAs with repeated-measures and Tukey’s HSD tests to investigate the influence on TTC and ART for driving safety [48] (except for the brake response time). Table 1 shows the means and standard deviations of the dependent variables. In cases where the participants were unable to react to avoid an event, the data from the accelerator and brake response times were eliminated. The distance between the host vehicle and the vehicle in the blind spot was recorded and analysed.

**Table 1:** Mean (*M*), standard deviation (*SD)* of the number of compensatory head movements, pedestrian collisions, the subjective ratings, time to collision, and accelerator response time for each condition.

	Baseline *M (SD)*	WC *M (SD)*	WoC *M (SD)*
**Head movements (Objective)**	23.78 (38.20)	132.11 (51.93)	1.31 (1.96)
**Head movements (Subjective)**	22.89 (35.34)	137.94 (47.23)	0 (0)
**Pedestrian collisions**	0.06 (0.24)	2.72 (2.19)	6.72 (2.91)
**Subjective ratings on difficulty**	1.83 (1.24)	4.72 (1.13)	6.33 (1.03)
**Time to collision (s)**	2.39 (0.52)	1.66 (1.12)	0.86 (1.16)
**Accelerator response time (s)**	1.76 (1.70)	2.78 (2.00)	4.94 (3.06)

For the analysis of brake response time, the number of patients who failed to cope with the pedestrian during driving was estimated by a survival analysis using the Kaplan–Meier method, which has been applied to estimate patients’ collision risk in follow-up studies (e.g. [49–50]). The data of drivers who did not push the brake pedal were not evaluated. There were several drivers who had collisions even though they pressed the brake pedal. These data were censored, while the data of drivers who pressed the brake and avoided pedestrian collisions were regarded as uncensored data (i.e. complete data). Statistical analyses were implemented in IBM SPSS software version 25.0 (SPSS Inc., Chicago, Illinois, USA) and R version 3.4.2 (R Core Team).

## Results

### Number of compensatory head movements

**Fig 5:**
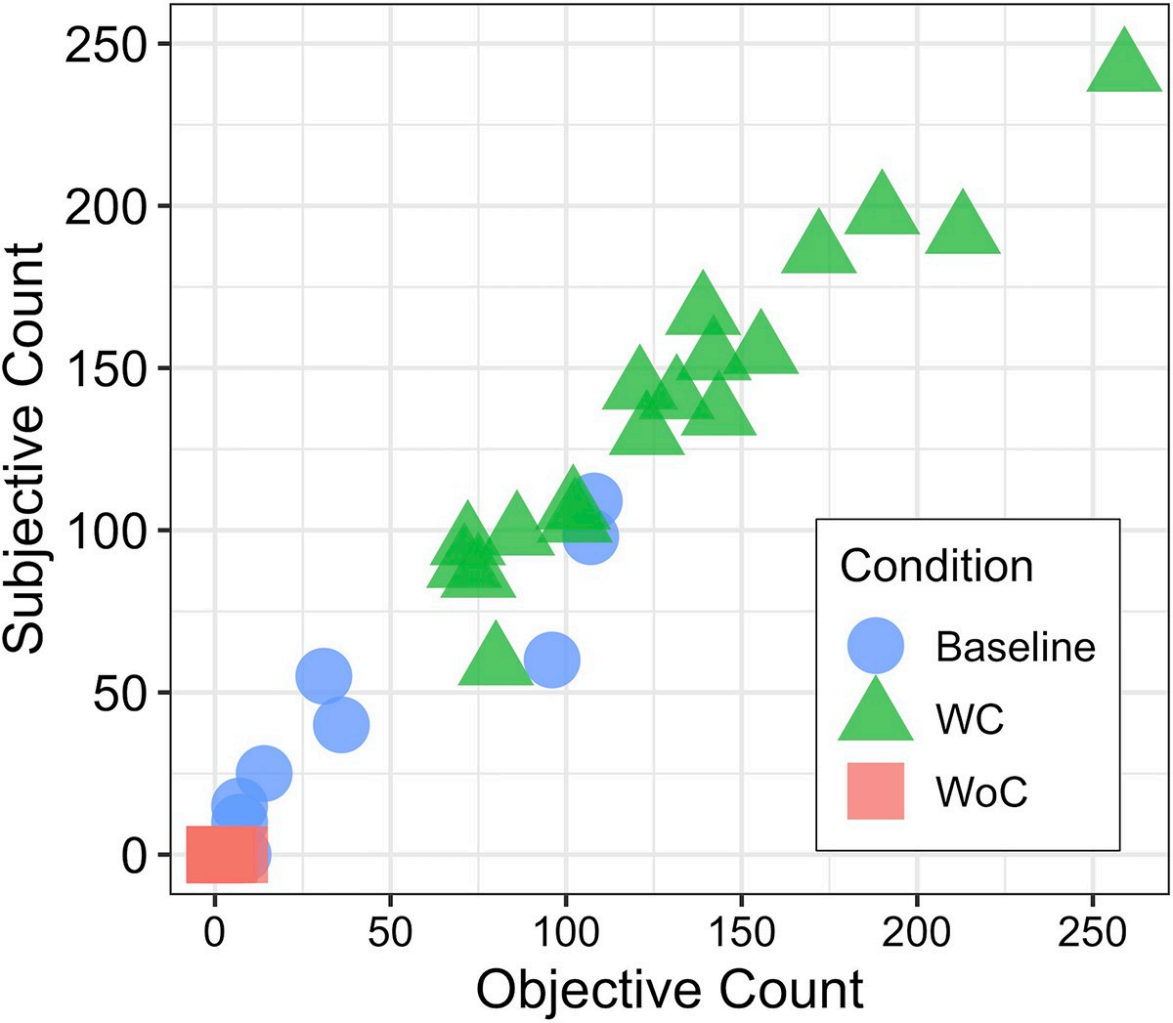
Scatter plot of the compensatory head movements for each condition.

Fig 5 shows a scatter plot of subjective and objective counts of compensatory head movements across three experimental conditions. There was a strong positive correlation between the two parameters in the Baseline, *r*(16) = 0.95, *p* < 0.001, range = 0–108 times, and the WC condition, *r*(16) = 0.96, *p* < 0.001, range = 71.5–213 times. Drivers performed compensatory head movements more under the WC than the Baseline condition (Table 1), paired-samples *t*(17) = 7.39, *p* < 0.001. The result shows that participants moved their head actively under the WC, indicating that participants appropriately followed the instructions.

The correlation analysis showed a negatively moderate correlation between the numbers of pedestrian collisions and counted head movements, objective count: *r*(16) = –0.45, *p* = 0.05; subjective count: *r*(16) = –0.50, *p* = 0.03. This result suggests that, in the WC, the increased amount of compensatory behaviour may make drivers avoid a surprise hazardous pedestrian. Two groups were divided based on the number of compensatory head movements: low-compensation group (bottom half; mean number = 92.5, SD = 20.37, range = 71–123) and high-compensation group (upper half; mean number = 171.72, SD = 42.26, range = 131.5–259). Independent-samples t-test examined difference of the number of compensation between low- and high-compensation group, *t*(16) = 4.6, *p* < 0.001. The mean number of collisions in the low-compensation group was greater than that of high-compensation group, however, independent-samples t-test revealed no significant difference of the number of collisions was observed between two groups, *t*(16) = 1.30, *p* = 0.21, *M* = 3.33 vs. 2.00.

### Time to collision

Table 1 illustrates mean and standard deviation values of TTC for each condition. A repeated-measures ANOVA found a significant main effect of TTC, *F* (2, 34) = 55.46, *p* < 0.001, η_G_^2^ = 0.59. Tukey’s HSD tests found significances among all conditions. The drivers with visual field defects performing compensation showed lesser collision risk than when they did not perform compensation under visual field defects (*p* < 0.001) and when they drove without visual defects (*p* < 0.001). However, the drivers with compensation also had a higher collision risk than drivers without visual field defects (*p* < 0.001).

### Driver perception of hazardous pedestrian

#### Accelerator release time

Table 1 illustrates mean and standard deviation values of ART for each condition. A repeated-measures ANOVA was also conducted to test the accelerator response time, *F*(2, 34) = 107.33, *p* < 0.001, η_G_^2^ = 0.76. Tukey’s HSD tests found significances among all conditions identical to TTC. The drivers’ reaction with the accelerator pedal was earlier when they performed compensatory behaviour than when they did not (*p* < 0.001). The drivers without visual field defects released the accelerator pedal in a timely manner compared to the two driving conditions with visual field defects, WC (*p* < 0.001) and WoC (*p* < 0.001).

#### Radius, distance between the vehicle and pedestrian, and compensation

The effectiveness of compensation in hazardous events was observed using three measures: radius, distance between the vehicle and the pedestrian on the X-axis, and the range of compensation in the WC condition. The time series of these three measures, speed, and the inputs of the accelerator and brake pedals were plotted for each participant and event in the WC. When the distance between the vehicle and the pedestrian on the X-axis was greater than the radius, the speed and input of accelerator pedal decreased after the driver moved their head towards the pedestrian. We considered this behaviour as compensation, and it was effective in avoiding traffic collisions. However, even if they did not show any compensation, drivers sometimes coped with the events.

For each condition, 180 event cases occurred (10 events * 18 drivers), and 48 events resulted in collisions while 132 were successfully avoided in the WC condition (see Table 2). According to the estimation using the aforementioned variables, 94 of the 132 cases avoided in the WC occurred with compensatory behaviour, while 38 occurred without it.

**Table 2:** Summary of Kaplan–Meier estimates for driver brake response time, and the number of accelerator and brake pedal operations, and collision-avoided and -involved for each condition.

	Mean survival time (s)	Accelerator released Number	Brake pressed Number	Uncensored Number (Collision-avoided)	Censored Number (Collision-involved)
**Baseline**	2.01	180	180	179	1
**WC**	3.22	146	146	132	14
**WoC**	5.62	108	104	58	46

### Brake response time

A survival analysis was performed using the Kaplan–Meier method. The total number and mean survival time are summarised in Table 2. All drivers pressed the brake at all events under the Baseline. However, some drivers did not push the brake at several events under the WC and WoC conditions.

**Fig 6:**
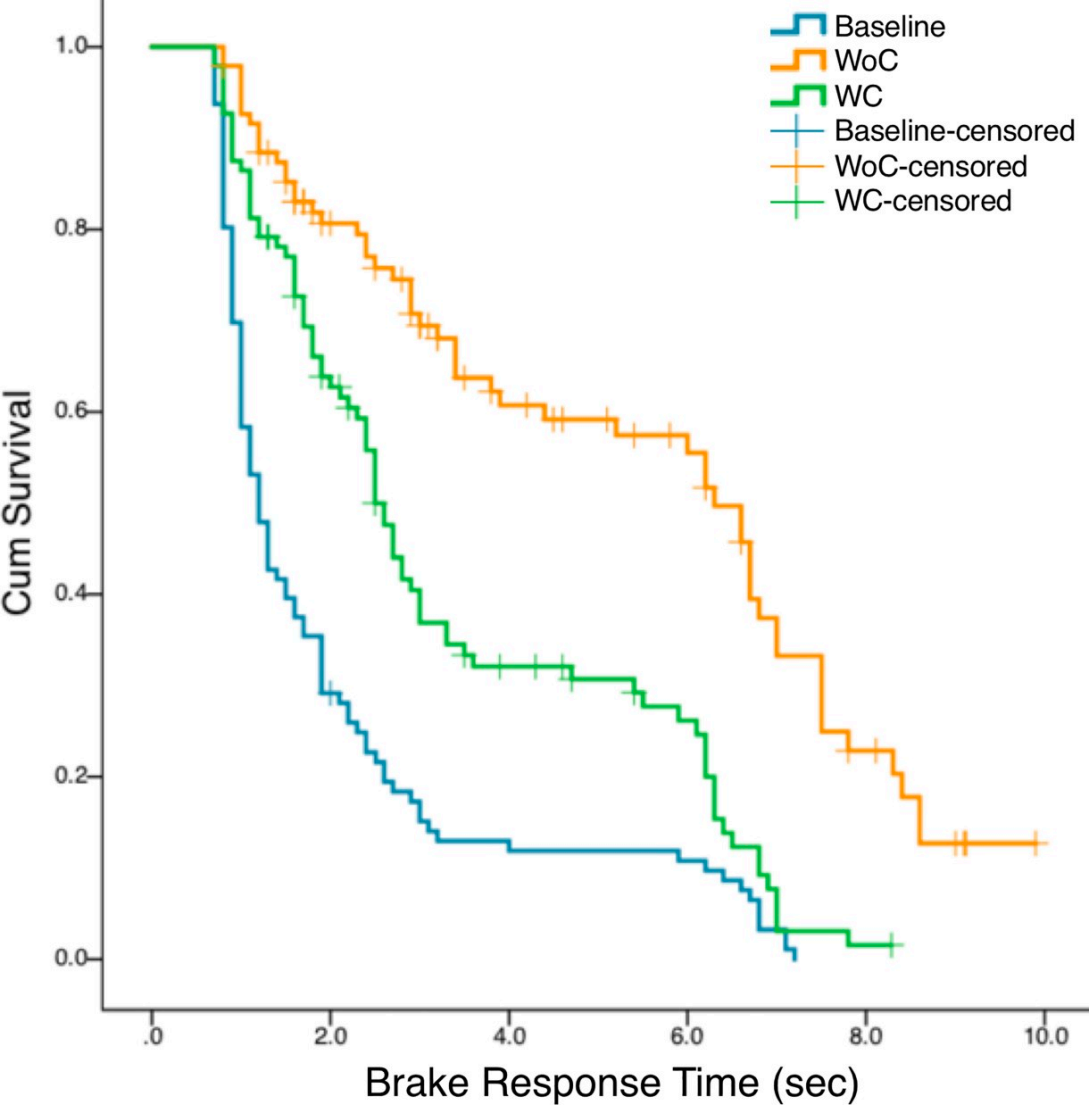
Kaplan–Meier survival curve estimating the proportion of pedestrian collisions relative to brake response time.

The Kaplan–Meier survival curves estimated the proportion of pedestrian collisions of driver brake response time for each condition (Fig 6). According to the overall comparison of survival curves using the Log Rank test, three survival curves significantly differed, χ^2^ = 115.56, *p* < 0.001. Pairwise comparisons using the Log Rank test observed significances among all conditions (*p* < 0.001). The results indicate that drivers reacted quicker in the Baseline compared to the WC and the WoC conditions. The estimated survival proportion was 0.02 and 0.13 in the WC and the WoC respectively.

### Number of collisions with pedestrians

The number of collisions with pedestrians was tested by a repeated-measures ANOVA, *F*(2, 34) = 61.16, *p* < 0.001, η_G_^2^ = 0.64. As shown in Table 1, the drivers involved in most collisions with a suddenly approaching pedestrian under the WoC. The number of collisions was greater in the WoC than the WC (*p* < 0.001). Compared to the Baseline, drivers had more accidents under the WC (*p* < 0.001) and WoC (*p* < 0.001). Unlike the Baseline, where drivers (with one exception) did not experience any collisions, under the WC, there was an average of approximately 3 collisions per driver.

### Subjective ratings on difficulty

A repeated-measures ANOVA was again used to assess participants’ subjective ratings of the difficulty of each driving condition, *F*(2, 34) = 110.27, *p* < 0.001, η_G_^2^ = 0.74. As shown in Table 1, most drivers reported that the WoC was the most difficult condition to drive. Pairwise comparisons revealed that the ratings of difficulty in the WoC were higher than those of Baseline and WC (*p*s < 0.001). Compared to the Baseline, drivers experienced difficulty driving under the WC condition (*p* < 0.001).

## Discussions

The main objective of this study was to investigate the effects of a driver’s compensatory head movements on avoiding pedestrian collisions under conditions simulating a constricted visual field. In this study, we simulated advanced visual field defects using a special eyeglass which narrowed the participants’ binocular visual field to approximately 10°. The driving simulator experiment with simulated advanced visual field defects involved two designs: with and without active compensatory head movement (condition ‘WC’ & ‘WoC’). These designs were compared to driving without visual field defects (‘Baseline’). Active exploratory compensation improved driving performance increasing traffic safety. However, significantly late response time and high collision rate compared to performance while driving without visual defects reflects limited effects of compensation. Overall, the current study found visual field defects are closely involved with decreases of hazard perception and recognition. Both effects and limits of compensation provide an important insight into how to assist traffic safety for visually impaired drivers.

Active exploratory behaviour leads reduced risk and rates of pedestrian collisions. In current study, we encouraged the participants make active and frequent head movements, and the counted number of compensatory showed that they appropriately followed our instructions. The analyses of response time resulted that both ART and BRT were the largest in the WoC among the three experimental conditions. Drivers under the WoC also require a longer time to operate the accelerator and brake pedals compared to the WC. The evaluation of TTC helped determine whether it is possible to reduce the risk of pedestrian collisions by applying active compensation. As expected, drivers showed superior performance under the WC than they did under the WoC condition, indicating that active compensatory behaviour has a greater potential to reduce traffic collision risk than driving without head movement. Lastly, we also confirmed whether compensation actually helped the collision reduction by observing three measurements, radius, distance between the vehicle and the pedestrian on the X-axis, and the range of compensation in the WC. The compensation led drivers to detect half of the all hazardous events during compensation. Because the present study simulated visual field defects for healthy-sighted drivers, estimating whether compensatory behaviour actually moved towards the hazard is relatively difficult in comparison with studies those tracked gaze-movements for visually impaired patients (e.g. [28, 40]). This method can be applied to further study concerning simulations of advanced visual defects as well as other traffic scenarios–e.g. oncoming-turning vehicles. In current study, statistical analysis was not conducted for the three measurement, thus, future study is expected to make quantitative analyses as combines behavioural measurement.

Reported trends in effects of compensatory behaviour on driving safety may be related to the frequency of compensation [24, 38–40]. The present finding that the collision number did not differ according to the frequency of compensatory head movements is inconsistent with the reported trend. There could be several reasons why our result differed from previous studies–e.g. drivers in an early stage of visual impairment or road environment. Individual differences, such as age or driving experience, may be likely to have impacts on the different trend. Our present finding confirms effectiveness of compensation in a limited situation as well as has an implication that the number of times drivers apply compensation is insufficient to indicate how effective this strategy is at reducing collisions compared to that of driving without visual impairment. Further research is needed to consider how to develop a comprehensive mechanism decreasing collision rates with respect to such differences.

It is worth mentioning that the potential of active compensatory viewing movements for drivers with tubular visual field disabilities is limited. In contrast to prior finding that binocular visual field defects do not have a substantial impact on driving safety (e.g. [36]), the current study found that drivers ignored 34 of 180 events in the WC, whereas drivers under the Baseline condition responded to all hazardous events (see Table 2). The finding indicates that even if drivers with advanced visual field disabilities make excessive compensatory head movements, it is difficult to overcome their impairment and raise the driving safety to the level of driving with an adequate visual field–i.e. late operation response or greater number of pedestrian collisions. The impairment to driving is likely to greatly dependent on the extent of the damage to the driver's visual field. As aforementioned, a future study is expected to look into differences that produced by degrees of visual impairment, for example, comparisons of driving performance between drivers in an early and advanced stage of visual defects.

Little research has addressed limiting compensation while considering drivers’ perception and recognition of traffic hazards, whilst the potential of compensatory behaviour has been suggested (e.g. [29, 32–33]). Hence, to clarify at which stage drivers have difficulty in coping with pedestrian collisions, driver perception and recognition of a hazardous pedestrian were estimated by analysing the ART and BRT. Releasing the accelerator pedal can be equated to a driver’s perception of a hazardous pedestrian, while pressing the brake pedal can be equated to the recognition of that hazard. In this study, there were no drivers who did not press the brake pedal after releasing the accelerator pedal under the Baseline and WC conditions, and this tendency was approximately identical to that of the WoC. However, under the WC and WoC, drivers did not release the accelerator to avoid several events, whereas drivers reacted to all events under the Baseline (see Table 2). Under the WoC condition, there were the greatest number of nonreactions when considering the accelerator (WoC = 72; WC = 34 events). This result reflects that visual field defects caused failures in hazard perception as well as active exploratory behaviour is likely to compensate for the deteriorated hazard perception by visual defects. In particular, analyses of BRT and the number of collisions also indicates that, when driving with visual impairment, late recognition of the pedestrian increases the likelihood of being involved in traffic collisions. To handle a dynamically changing road environment, it is important to perceive the presence of a traffic hazard and appropriately recognise it as a hazardous object. Our present finding implies that the behavioural consequences upon the failure of hazard perception determines involvements in pedestrian collisions for drivers with advanced visual field defects.

Analyses of the two response times imply that if assistance of hazard perception for visually impaired drivers is provided, the drivers are likely to have better and appropriate perception as well as consequent recognition of traffic hazards. Glaucoma is a leading cause of blindness [51], and the symptoms develop slowly. Optic diseases related to visual field like glaucoma are not treatable by medicine and are closely related to aging. Aging glaucoma patients are more associated with driving cessation and involved in motor vehicle collisions than age-matched individuals without the optic disease [52–53]. Regarding this problem and a patient’s tendency to drive daily without self-awareness of their symptoms [8], appropriate machinery assistance could improve their driving safety. Our findings suggest that drivers require machinery assistance to support appropriate situation awareness. In addition, the rapidly developing field of driving automation may also offer benefits for such drivers. Further research may be needed to clarify which types of supportive system would be most effective to assist their driving safety.

The current finding should be interpreted with at least four limitations. First, the driving scenarios for each condition included similar events, which poses problems for comparability between conditions. The design of events was also not based on real cases of typical serious pedestrian collisions. Future studies, therefore, must design experiments with identical scenarios that include unified and calculated pedestrian hazardous events to examine the effects of compensation more precisely. Second, this study regarded only pedestrian collisions rather than several types of motor vehicle collisions. In related vein, longitudinal controls were implemented in the current study unlike a real-world driving which requires comprehensive operations of longitudinal as well as lateral controls. In future studies, in addition to pedestrian collisions from the sides, it would be useful to examine motor vehicle collisions with complicated controls with respect to natural driving situations. Third, the experimental task required drivers to make an extended effort that was unnatural and would not occur in typical on-road driving. Further studies should regard the drivers’ working capacity (e.g. [54]). Lastly, a previous study has noted the limitations of studies without actual patients; drivers simulating visual field defects were used instead [2]. There may be negative impacts on driver behaviour in our repeated-measures design study. Future study should consider alternative methods to examine different compensatory behaviour, such as eye movement,–e.g. gaze-contingent software that shows a mask on the screen of simulator. In a related vein, the extent of visual field loss for actual patients is perhaps associated with types of traffic accidents as well as levels of injury severity. Therefore, clarifying factors affecting their injury severity in a variety of traffic situations could be highlighted in future study (e.g. [55–57]). However, there is published research that describes drivers’ hazard detection through the simulation of visual field defects or modelling (e.g. [28, 58]). Future study should explore the behaviour of visually defected drivers with true patients or other apparatus that can simulate visual field defects more naturally.

## Conclusions

In a driving simulator experiment involving a special eyeglass to imitate advanced visual field defects, 18 participants experienced three driving conditions (Baseline, WC, and WoC) to assess the effectiveness and limitations of compensatory head movements for dealing with pedestrian collisions. As hypothesised, in comparison with the driving performance in the WoC, active compensation enabled visually impaired drivers to avoid more collisions with the hazardous pedestrian from the left of the driving lane, improving their ability to detect a traffic hazard. However, even though drivers attempted to make active compensatory movements under the WC, there were several unavoidable hazards, whereas they recognised and reacted to all hazardous events under the Baseline. Thus, this study indicates that, while compensation can reduce the collision risk to some degree, it has limitations and is not able to enhance either the driving performance or hazard perception to the level of driving without visual field impairment. To reduce the risk of traffic accidents, designing machinery assistance for such drivers should be considered in the future. Future research should also address the practical application of compensatory viewing strategies in the real-world.

## Supporting information

S1 Dataset (XLSX)
